# Universal Features in Phonological Neighbor Networks

**DOI:** 10.3390/e20070526

**Published:** 2018-07-12

**Authors:** Kevin S. Brown, Paul D. Allopenna, William R. Hunt, Rachael Steiner, Elliot Saltzman, Ken McRae, James S. Magnuson

**Affiliations:** 1 Department of Biomedical Engineering, University of Connecticut, Storrs, CT 06269, USA; 2 Department of Physics, University of Connecticut, Storrs, CT 06269, USA; 3 Institute for Systems Genomics, University of Connecticut, Storrs, CT 06269, USA; 4 Connecticut Institute for the Brain & Cognitive Sciences, Storrs, CT 06269, USA; 5 Department of Psychological Sciences, University of Connecticut, Storrs, CT 06269, USA; 6 Department of Physical Therapy and Athletic Training, Boston University, Boston, MA 02215, USA; 7 Department of Psychology, University of Western Ontario, London, ON N6A 5C2, Canada; 8 Brain & Mind Institute, University of Western Ontario, London, ON N6A 5C2, Canada

**Keywords:** networks, neighborhood activation model, phonology, phonological neighbor network

## Abstract

Human speech perception involves transforming a countinuous acoustic signal into discrete linguistically meaningful units (phonemes) while simultaneously causing a listener to activate words that are similar to the spoken utterance and to each other. The Neighborhood Activation Model posits that phonological neighbors (two forms [words] that differ by one phoneme) compete significantly for recognition as a spoken word is heard. This definition of phonological similarity can be extended to an entire corpus of forms to produce a phonological neighbor network (PNN). We study PNNs for five languages: English, Spanish, French, Dutch, and German. Consistent with previous work, we find that the PNNs share a consistent set of topological features. Using an approach that generates random lexicons with increasing levels of phonological realism, we show that even random forms with minimal relationship to any real language, combined with only the empirical distribution of language-specific phonological form lengths, are sufficient to produce the topological properties observed in the real language PNNs. The resulting pseudo-PNNs are insensitive to the level of lingustic realism in the random lexicons but quite sensitive to the shape of the form length distribution. We therefore conclude that “universal” features seen across multiple languages are really string universals, not language universals, and arise primarily due to limitations in the kinds of networks generated by the one-step neighbor definition. Taken together, our results indicate that caution is warranted when linking the dynamics of human spoken word recognition to the topological properties of PNNs, and that the investigation of alternative similarity metrics for phonological forms should be a priority.

## 1. Introduction

### 1.1. Background

The perception and recognition of acoustic speech, known in psycholinguistics as *spoken word recognition* (SWR), requires that human listeners rapidly map highly variable acoustic signals onto stable linguistically relevant categories (in this case, phonemes, i.e., the consonants and vowels that comprise a language’s basic sound inventory) and then piece together sequences of phonemes into words, all without robust cues to either phoneme or word boundaries (see [1,2] for reviews). Decades of research on human spoken word recognition have led to a consensus on three broad principles: (1) SWR occurs in a continuous and incremental fashion as a spoken target word unfolds over time; (2) words in memory are activated proportionally to their similarity with the acoustic signal as well their prior probability (computed as a function of their frequency of occurrence) in the language; and (3) activated words compete for recognition. A key difference between theories is how to characterize signal-to-word and word-to-word similarity. Most theories incorporate some sort of similarity threshold, and pairs of words meeting that threshold are predicted to strongly activate each other and compete. Perhaps the most influential definition for the phonological similarity of spoken words is the concept of phonological neighbors posited under the Neighborhood Activation Model (NAM) by Luce and colleagues [3,4]. NAM includes a gradient similarity metric and a threshold metric, although only the latter is widely used (and we focus on it here). The threshold metric defines neighbors based on the Deletion-Addition-Substitution (DAS) string metric, which states that two words are neighbors (i.e., they are sufficiently similar to strongly activate one another and compete) if they differ by no more than the deletion, addition, or substitution of a single phoneme. Thus, *cat* has the deletion neighbor *at*, addition neighbors *scat* and *cast*, and many substitution neighbors, such as *bat*, *cot*, and *can*. NAM predicts that a target word’s recognizability is determined according to a simple *frequency-weighted neighborhood probability rule* which is defined by the ratio of the target word’s prior probability to the summed prior probability of all its DAS-linked neighbors. The NAM rule predicts a greater proportion of the variance in spoken word recognition latencies (10–27%, depending on task [lexical decision, naming, or identification in noise] and conditions [signal-to-noise ratio] [3]) than any other measure that has been tested (e.g., log word frequency alone accounted for 5–10% of variance in Luce’s studies).

The focus of the NAM approach has typically been to characterize the recognizability of single words according to the sizes (densities) of their locally defined neighborhoods. More recently, it has been realized that viewing the structure of the phonological lexicon globally as a complex network enables the probing of connections between both large and small scale network topology and human spoken word recognition. Thus, rather than considering a word and its neighbors in isolation, the set of neighbor relationships for an entire lexicon can be represented as an unweighted, undirected graph [5] in which words (phonological forms) are represented by nodes and two words are joined by an edge if they meet the standard NAM DAS threshold. The NAM approach can be translated to the network context to mean that (frequency-weighted) node degree is important for predicting latencies in spoken word recognition. There are also prior indications that other topological properties (e.g., the clustering coefficient [6,7], closeness centrality [8], and second neighbor density [9]) may also explain some aspects of SWR that the frequency-weighted neighborhood probability it is based upon does not.

Previous studies have shown that what we will call the *phonological neighbor network*, or PNN, for English has some features of both Watts-Strogatz [10] and Barabasi-Albert [11] graphs. It has a relatively short mean geodesic path length and high clustering coefficient, but also has a degree distribution that is at least partially power law [5]. Subsequent analyses of additional languages (English, Spanish, Hawaiian, Basque, and Mandarin) have shown these characteristics to be broadly shared across languages when PNN graphs are constructed using NAM’s DAS rule [12]. On the basis of these results, Vitevich and colleagues have assigned importance to these language “universals” and argued that many of these properties are sensible if not essential (e.g., high degree assortativity, which measures the tendency of nodes to be connected to other nodes of similar degree, can buffer against network damage) [12] (Consistent with the idea that these properties may be universal, networks connected based on part-whole relations between word forms [13] and networks connected based on semantic relations [14] exhibit similar properties. However, while the similarity across these networks is intriguing, we think skepticism about deep universals is warranted pending deeper analysis. Similarities across these networks is most salient in degree distributions, which appear consistent in each case with relatively scale-free networks. As we discuss below, for the case of phonological networks, scaling parameter estimates are quite sensitive to analysis decisions such as whether or where to truncate distributions. It is important in each case to determine the relative importance of constraints imposed by connecting rules vs. analysis decisions vs. intrinsic aspects of language in determining network characteristics (that is, understanding why a particular network has certain characteristics). In this paper, we restrict our scope to the most prominent approach to phonological networks in the literature, the DAS rule from the NAM, and leave similar consideration of other approaches for future research).

However, making claims about SWR on the basis of the properties of PNNs alone is potentially fraught for at least two reasons. First, PNNs are static representations of lexical structure, whereas spoken words are processed incrementally over time. Second, different measures of word similarity will result in radically different PNNs. NAM’s DAS rule is based on a relatively simple string distance metric that provides a local measure of inter-word similarity that is insensitive to the sequence of phonemes in a word. Thus, while NAM’s DAS metric accounts for substantial variance using a regression-based approach (predicting response latencies for many words), there is substantial evidence from studies examining competition between specific pairs of words with different patterns of position-dependent phonological overlap that words whose onsets overlap compete more strongly than words that are matched in DAS similarity but whose onsets are mismatched (e.g., *battle* would compete more strongly with *batter* than with *cattle* [15]). Marslen-Wilson and colleagues [16,17] proposed a threshold metric that gives primacy to onset similarity. They focused on the notion (consistent with many priming and gating studies [17]) that the “cohort” of words activated by a spoken word is restricted to words overlapping in their first two phonemes. Thus, the cohort competitors of *cat* include not just DAS neighbors overlapping at onset (*can*, *cab*, *cast*) but also longer words that would not be DAS neighbors (*cattle*, *castle*, *cabinet*). In addition, the cohort metric predicts that rhyme (i.e., a word’s vowel and following consonants) neighbors (*cat-bat*, *cattle-battle*) do not compete because they mismatch at onset, despite high DAS similarity. A PNN based on a simple onset cohort rule (connect words that overlap in the first two phonemes) would obviously have very different structure than a DAS-based PNN. When using PNNs to compare lexical structure between languages, we must consider the potential role of the similarity metric itself in determining the network’s structure and topology. This possibility calls into question any universal (language-independent) claims about SWR based on DAS networks. Prior work has demonstrated that this is likely true at least in English, as PNNs constructed from a random lexicon with the same phonological constraints as English are basically indistinguishable from the real language network [18,19].

### 1.2. Hypotheses

Based on the discussion above and prior work in English [18,19], we have the following hypotheses. First, we believe that the one-step DAS neighbor rule will produce phonological neighbor networks that look very similar to those for real languages, even if the set of strings to be connected has only some of the features of a real lexicon. Second, we hypothesize that the strong contraints on connectivity induced by the DAS rule give rise to the observed “universal” topological features seen across multiple languages [12]. Third, we believe that the topological properties of a PNN will be form-length dependent. That is, a PNN constructed of only short (monosyllabic) words will have different properties from a PNN constructed from long (polysyllabic) words. The observed PNN degree distribution will therefore be a mixture distribution of size-class specific distributions.

## 2. Results

### 2.1. Empirical Analysis of Phonological Neighbor Networks

#### Degree Distributions and Topology

**Figure 1:**
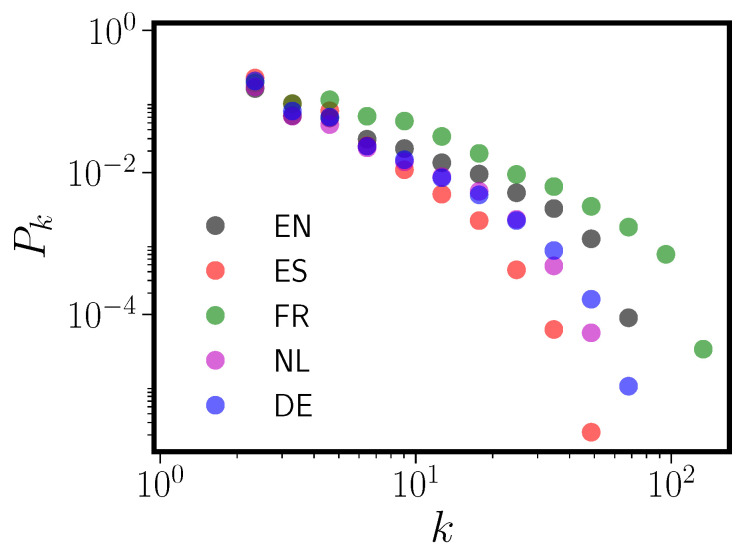
Log-log plot of the logarithmically binned phonological neighbor network (PNN) degree distribution $P_{k}$ for five languages: English (EN), Spanish (ES), French (FR), Dutch (NL), and German (DE).

Figure 1 shows the degree distributions for the five PNNs constructed from the CLEARPOND data (see Section 5.1 for information about CLEARPOND; compare also to Figure 10 in the original CLEARPOND paper [20]), and Table 1 gives a summary of some of the common topological measures employed in the empirical analysis of networks, all of which have been specifically highlighted in prior PNN research. All five language degree distributions are best fit (via maximum likelihood) by a truncated power law, as tested via likelihood ratio [21]. In addition, we observe that all PNNs have: (i) relatively high clustering; (ii) short mean geodesic paths; (iii) extraordinarily high values of degree assortativity; and (iv) relatively small giant connected components (the largest connected subgraph in the network). Thus, all five PNNs have similar degree distributions and topological characteristics, and they combine some features of Watts-Strogatz [10] graphs (high clustering) with Barabasi-Albert graphs [11] (power law degree distribution). High degree assortativity and small giant component sizes are features of the PNNs that are not displayed by either WS or BA graphs. These features are all consistent with previous studies on English alone [5,18] and other languages not studied here [12].

The grouping of languages in Figure 1 is rather surprising. Essentially, Spanish is by itself, Dutch and German have quite similar degree distributions, and English and French are grouped together. One might expect different clustering based on language typology; for example, with the two Romance languages (French and Spanish) grouped together. We will show that the observed clustering can be explained without any reference to the specific history of words. Instead, the structure of the phonological form length distribution, along with target language phoneme frequencies, are all that is required.

### 2.2. Islands and Frequency Assortativity

**Figure 2:**
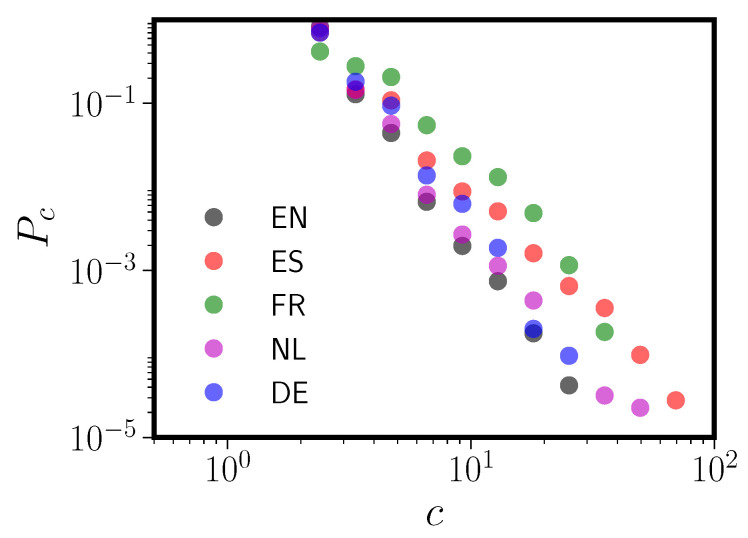
Size distributions $P_{c}$ vs. *c* for the connnected components (“islands”) in all five languages. Languages are abbreviated using their two-letter ISO codes (see Table 1). The giant component has been excluded from this figure for all five languages; it sits far to the right for each language. The minimum island size is two because we have removed any singleton nodes (loners) from the PNNs, as discussed in Section 5.1.

Given the relatively modest size of the giant connected component in all five languages (see Table 1), it is worth examining the connected component size (“island size”) distribution $P_{c}$ for each of the five PNNs. Power law distributions for the sizes of the connected components $P_{c}$ have been previously observed in PNNs for both English and Spanish [22]. Figure 2 shows that this power law distribution of component sizes is broadly shared over all five languages. In fact, the island size distribution is more robustly power law than the PNN degree distribution itself, albeit over a relatively modest range (less than a factor of 100).

We now remark on a previously unobserved feature of PNNs, again present in all five languages. All five languages show a weak but statistically significant degree of word-frequency based assortativity. Simply, words of similar usage frequency tend to be connected to each other in the PNN. We computed frequency assortativity by dividing the continuous word frequency data into ten equal-mass bins and then computing an assortativity coefficient and jackknife standard deviation using the definitions in Newman [23]. The values ranged from 0.1 in English to 0.24 in Spanish, which correspond to between 26 and 47 Jackknife standard deviations. This is weak relative to degree assortativity in these networks (see Table 1), but not insignificant on the scale of assortativity coefficients found in other social, biological, and technological networks [23]. Earlier work in English [24], not based on network analyses, found a positive correlation between the number of neighbors of a word and its frequency. Frequency-based assortativity is not simply a degree-frequency correlation, as it indicates words of similar frequency tend to be connected to one another, regardless of degree. However, each PNN also shows high degree assortativity (see Table 1), and insofar as frequency and degree may be correlated, the two types of assortativity may be linked. Disentangling this relationship is beyond the scope of the current study.

### 2.3. DAS Graphs as Mixtures

There is a deep physical basis for observing power laws in thermodynamics. Diverging length scales at critical points mean that there are correlations at all scales in the system. Critical point behavior cannot depend on any quantity (like a force) with an associated length scale, but rather only on scale-free quantities like symmetries and conservation laws. Critical point phenomena then become universal, in the sense that the same behavior (critical exponents) is observed in systems that may have radically different forces but the same set of symmetries.

The converse is not true. Observation of power laws does not necessarily indicate any deep phenomena at work. Power laws in empirical data can arise from a wide variety of reasons, many of them mundane. One of the simplest is Simon’s famous demonstration [25] that multiplicative (rather than additive) random noise can yield heavy-tailed distributions. Another way to obtain power laws is via mixture distributions; in this case apparent scale-free behavior arises by simply mixing several distributions, each with well-defined but different scales.

**Figure 3:**
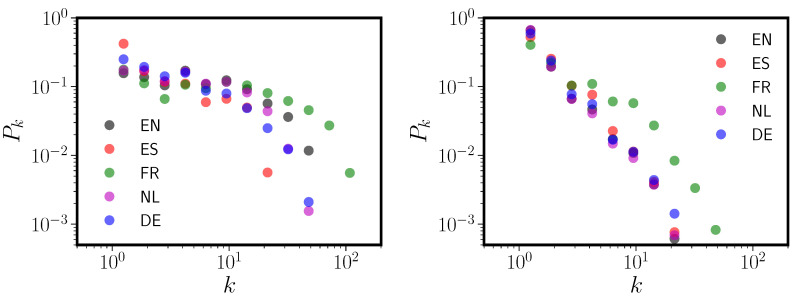
Degree distributions $P_{k}$ vs. *k* for PNNs formed from exclusively monosyllabic (**left panel**) or polysyllabic (**right panel**) words in each lexicon. Each language is abbreviated by its two letter ISO code; see the caption to Table 1 for the key to these codes.

Indications that the degree distribution of the PNN for English results from a mixture of distributions of different scales have been advanced by others [18]. Degree distributions for English PNNs separately constructed from short and long (in phonemes) words showed different shapes and, at least for short words, displayed markedly less power-law behavior. In Figure 3 we show that this result also holds for the CLEARPOND English corpus, as well as for Dutch, German, Spanish, and French. We divided all words in each corpus into two classes: monosyllabic and polysyllabic.

Figure 3 clearly shows that connectivity among only monosyllabic words differs from polysyllabic word connectivity. The monosyllabic degree distributions look less like power laws than do the polysyllabic degree distributions, and monosyllabic words are in general more densely connected than are polysyllabic words. This raises the possibility that the PNN degree distribution may arise as a mixture of distributions. In all five languages, networks formed from polysyllabic words have degree distributions that are much closer to (truncated) power laws than are the monosyllabic word networks. In addition, note that (with the exception of French) the polysyllabic degree distributions are much more similar across the five languages than the monosyllabic graph degree distributions or those of the full graphs (see Figure 1).

In Appendix A, we look more closely at phonological neighbor graphs formed exclusively from monosyllabic or polysyllabic words, and compare them to graphs containing all words in each corpus (see Table A1). We found that some of the full PNN topological properties are present in both the monosyllabic and polysyllabic networks (e.g., degree assortativity and the clustering coefficient). However, others are markedly different or disappear. The component or “island” size distribution $P_{c}$ is driven entirely by the polysyllabic words; the monosyllabic words are almost completely connected (an unsurprising outcome of the DAS rule; shorter words, such as *cat*, are much more likely to have DAS neighbors than long words like *catapult*). The full PNN graphs have short (∼7) average path lengths primarily because the monosyllabic graphs have extremely short average path lengths (∼5) and the polysyllabic graphs have long (∼10) ones. When we compare the local properties of the monosyllabic words in both the monosyllabic and full graphs, numbers of neighbors and second neighbors are highly correlated. However, clustering is more weakly correlated, indicating that explanations of latencies in SWR that appeal to node clustering [6] coefficient as a predictor may be quite sensitive to whether or not polysyllabic words were included as items in the experiment. Figure 3 and Table A1 confirm our second hypothesis, which is that the topologies of the monosyllabic and polysyllabic PNNs are different, and that the full PNN is a mixture of multiple size-class specific networks.

At least three questions remain. First, do constraints imposed by the one-step neighbor DAS similarity measure explain the apparently universal topological features seen across all five languages? If so, what explains the observed differences in the degree distributions in Figure 1? Finally, how much lexical structure is required to generate PNNs that resemble those of real languages? In what follows, we address these three questions in detail.

## 3. Pseudolexicons

**Figure 4:**
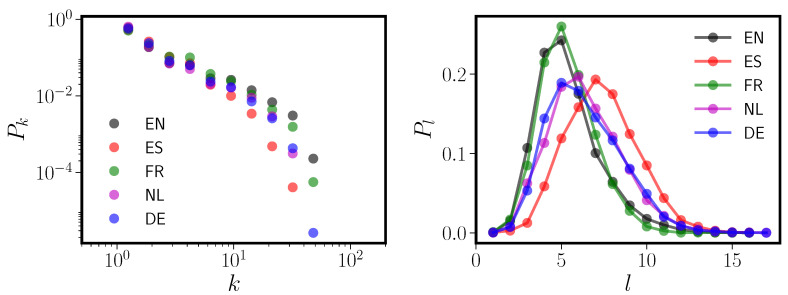
The left panel shows the degree distributions $P_{k}$ versus *k* for the five CLEARPOND PNNs. Compare to Figure 1; this figure differs because homophones have been removed from the graphs as detailed in Appendix B. The right panel shows the distribution $P_{l}$ of phonological form lengths in each of the five languages from the CLEARPOND corpora. Please note that all these distributions are only defined for l≥1; length zero words do not exist.

Figure 3 and additional results that we present in the Appendix A suggest that the truncated power law behavior observed in the five PNNs might be the result of mixing subgraphs with different connectivity properties. The left panel of Figure 4 again shows the degree distributions for the five languages, this time with all homophones removed. We discuss homophones in detail in Appendix B; in brief, we remove homophones because our random lexicon models produce phonological forms (rather than written words) directly and cannot properly account for homophones. The right panel shows the distribution $P_{l}$ of words of length *l* phonemes. The $P_{l}$ distributions are underdispersed relative to Poisson (not shown); note also that they are all zero-truncated, as there are no words in any language that consist of zero phonemes. A particularly intriguing feature of the five language $P_{l}$ is that they cluster similarly to the degree distributions shown in Figure 1. English and French are together, then German and Dutch, and Spanish by itself. This suggestive correspondence between the PNN degree distribution and $P_{l}$ for the five languages under study led us to refine one of our hypotheses in Section 1.2: our refined hypothesis is that one of the important linguistic inputs determining the PNN’s properties is $P_{l}$. While this correspondence between network and $P_{l}$ could be entirely coincidental or a result of previously undetected cross-linguistic similarities, below we will show that it is not.

### 3.1. Models

To determine which topological features of the PNNs arise due to specific features of real languages and which are driven purely by the DAS connection rule, we adopt and extend an approach inspired by previous work on the English PNNs [18]. We generate corpora of random phonological forms using generative rules that include varying amounts of real linguistic detail. We denote such a corpus of random strings of phonemes a pseudolexicon. Each pseudolexicon is paired with a target language, since all the models use some information from the real language for construction. Specifically, pseudolexicons are created from the phonemic inventory of each language (the set of all phonemes [consonants and vowels] that occur in the language), with lexicon size constrained to be approximately the same as the real-language lexicon for the target language (for example, about 22,000 unique words [i.e., excluding homophones] for English CLEARPOND), and with the same form length distribution as the target language. To match the length distribution, the length of each random string is first specified by drawing a random integer from a form length distribution $P_{l}$ defined on the positive integers excluding zero. In all cases, the pseudolexicon has a form length distribution which we specify. Specifically, we consider the following six models for pseudolexicons. We have named the models using terminology taken from the Potts [26] and Ising [27] models. Each includes progressively greater language-specific detail relating to phonological structure. We expected that we would get a successively better match to a given target-language PNN as we included more detail.

- **Infinite Temperature (INFT)**. Each phoneme in the string is drawn uniformly from the target language’s phoneme inventory. This model has no information about relative consonant (C) and vowel (V) frequencies in the target language; all are treated as equally likely.
- **Noninteracting, Uniform Field (UNI)**. Each phoneme in the string is drawn randomly using its observed frequency in the real language’s lexicon. This model receives information about overall C and V frequencies; for example, it is given the relative likelihood of finding /k/vs./a/ in English words. However, no positional frequency information is provided, so, for example, if asked to produce a three phoneme English pseudoword, UNI is no more likely to produce a CVC (a very common three phoneme pattern [/kæt/]) than a CCC (vanishingly rare, with some controversy regarding their status as actual words [/pst/]).
- **Noninteracting, Consonant/Vowel Uniform Field (CVUNI)**. Each position in the random string is either a C or a V drawn randomly using observed positional C and V frequencies in the real lexicon. Specifically, we use the real language’s corpus to compute the position-dependent probability that position *l* is a C or a V. The particular consonant or vowel placed at that position is drawn uniformly from the target language’s list of consonants and vowels. Unlike UNI, CVUNI would produce CVC more often than CCC if asked to generate a three-phoneme English pseudoword. However, the model is provided no knowledge of individual consonant and vowel frequencies, so common and uncommon phonemes will be mixed.
- **Noninteracting, Consonant/Vowel Field (CV)**. Positions are selected to be consonants or vowels exactly as in CVUNI. The particular consonant or vowel placed at each position is selected using observed frequencies of consonants and vowels from the real lexicon.
- **Noninteracting, Spatially Varying Field (SP)**. Each phoneme is drawn randomly from real positional frequencies in the target lexicon. For example, if a language has an inventory of twenty phonemes, we use the real lexicon to compute a $\pi_{l,x}$ that gives the probability that phoneme *x* occurs at position *l*, and then use this table to assign a phoneme to each position of the random string. SP and CV use similar but not identical information from the real language. One important feature of a real language that they do not capture is phonotactic constraints. That is, pairs of phonemes occur in real languages with frequencies different from the product of the frequencies of the individual phonemes, and in a word form location-dependent manner. For example, /t/ and /b/ are common consonants in English, but the diphone /tb/ rarely ever appears except in multisyllabic words at syllable boundaries (i.e., the words *outbreak*, *outburst*, *frostbite*).
- **Nearest Neighbor Interactions (PAIR)**. The first phoneme in each string is drawn using a positional probability. Subsequent phonemes are drawn via the following rule. If the phoneme at position *k* is *x*, then the phoneme at position k+1 is drawn using the empirical probability (from the real lexicon) that phoneme $x^{\prime }$ follows phoneme *x*. PAIR is the model we consider with the most (though not full) linguistic detail; unlike any other model above, PAIR will not produce unobserved diphones even if the constituent phonemes are quite common.

We have listed the models in rough order of complexity; INFT uses the least amount of information about the real language’s structure and PAIR the most. We note that while it is possible to generate real words (particularly short ones) from the models above, the vast majority of the strings produced bear no resemblance to real words in any of the five languages. The only model that avoids unpronounceable diphones is PAIR; in the other models unpronounceable diphones occur frequently.

**Figure 5:**
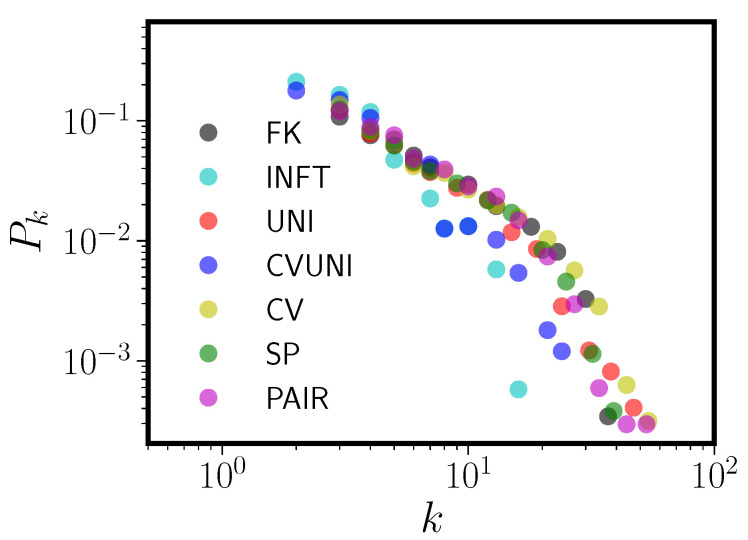
Degree distributions $P_{k}$ versus degree *k* for the Francis and Kucera 1982 corpus (FK) along with the six pseudolexicons fit to it. See the text for a key to the abbreviations for the pseudolexicons.

For each pseudolexicon, we discarded any duplicate items. This is why we removed homophones from the real languages; we did not generate orthographic tags for the random phonological forms, so duplicated forms in the pseudolexicon all represent a single node. We then formed a pseudo-PNN by using the DAS rule to connect items in the pseudolexicon to one another. As with the real PNNs, before any analysis we discarded nodes in the pseudo-PNNs with degree zero. Figure 5 shows the degree distribution of the Francis and Kucera 1982 English corpus (FK) [28] and its six corresponding pseudolexicons. We first show the fit to FK, rather than CLEARPOND English, due to our ability to better control the contents of the FK corpus (see Appendix B for details). Each of the six pseudolexicons had as its input $P_{l}$ the empirical English $P_{l}$ (e.g., Figure 4, right panel). We note that, while the sizes of the pseudolexicons were fixed to the real-language target lexicon, once the pseudonetworks are formed, they may have fewer nodes than this, since many pseudowords may be neighborless and hence not appear in the graph.

### 3.2. English Networks

To make a quantitative comparison of the degree distribution of a pseudo-PNN to that of its target language PNN, we calculate the Jensen-Shannon Divergence (JSD), defined as

(1) $$\mathrm{JSD}\left(P∥Q\right)=\frac{1}{2}D\left(P∥M\right)+\frac{1}{2}D\left(Q∥M\right),$$

where *P* and *Q* are discrete probability distributions, *M* is a mixture distribution (P+Q)/2, and $D\left(A∥B\right)$ is the Kullback-Leibler divergence between *A* and *B*. The JSD is symmetric in *P* and *Q*, always finite and (using base 2 logarithms in the KL divergence) bounded between 0 and 1. Figure 5 shows that even minimal levels of linguistic realism yield a pseudo-PNN with a stikingly similar degree distribution to the real English PNN, and the JSD in Table 2 bears this out. While more lingustic realism does (as expected) lead to a closer match to real English, the gains are not very large. Even UNI, which includes nothing beyond overall phoneme frequencies and the empirical $P_{l}$, is quite similar to FK. Table 2, which lists the same topological properties we previously showed in Table 1, tells an even more compelling story. First, the putatively lingustically relevant topological properties discussed earlier—high clustering, short mean path length, high degree assortativity, and (to some extent) small giant components—are present in all of the pseudolexicons whose degree distributions match that of FK. Giant component size is the least well-matched property in all of the models, though it is still smaller than observed in many real-world networks. Furthermore, even INFT, in which degree distribution (and hence mean degree) is a poor match to FK (largest JSD), has high clustering, short mean path length, and high degree assortativity. INFT includes almost nothing about the target language except the form length distribution and the phoneme inventory. We also note the noisiness in the degree distribution of INFT. While one might hypothesize that this is a result of its relatively small size, the degree distribution of INFT does not become smooth even for larger (10,000 node) graphs (not shown). While we hypothesized in Section 1.2 that the observed topology of PNNs constructed using the DAS rule could be obtained with less than full linguistic realism, we were surprised by how little of the structure of the real language is necessary to obtain a network that looks very much like that of a real language.

**Figure 6:**
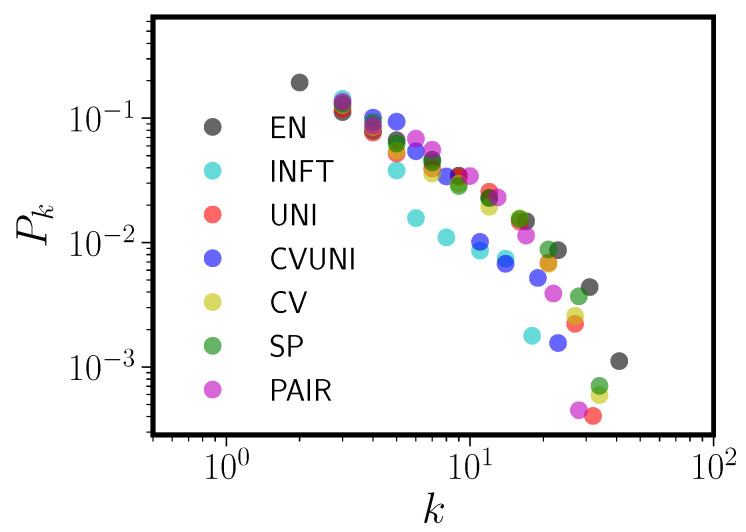
Degree distributions $P_{k}$ versus degree *k* for the CLEARPOND English corpus (EN) along with the six pseudolexicons fit to it. See the text for a key to the abbreviations for the pseudolexicons.

Figure 6 and Table 3 shows the same information for the CLEARPOND English database and pseudolexicons matched to it. We note first that all the conclusions that held for FK hold for CLEARPOND English. In fact, in the case of CLEARPOND, the JSDs between model and English are, with the exception of the poorly fitting INFT, all quite similar. In this case, UNI and CVUNI (surprisingly) are a better match than PAIR, the most detailed model. As before, UNI has a very similar degree distribution to the real English PNN and very similar topological characteristics. INFT, again despite having a degree distribution that is an extremely poor match to English CLEARPOND (worst match by JSD), has high clustering coefficient and high degree assortativity. Compared to FK, some differences are evident. Chiefly among them is that all the models now have too low of a mean degree, arising because the model degree distributions have large-*k* tails that are too short. However, given the analysis and discussion in Appendix B, this is to be expected. As discussed there, our models do not include analogs to inflected forms (e.g., *walk*, *walks*, *walked*). We also have not attempted to model homophones (which have been removed in our pseudo-lexicon PNNs) or proper nouns. All three of these item types preferentially affect the tail shape of $P_{k}$. We also note that the CLEARPOND-matched pseudolexicons tend (except for SP) to undershoot the English giant component size, though they still match the fundamental observation that the GC is a relatively small portion of the full network.

### 3.3. Five Language Pseudonetworks

**Figure 7:**
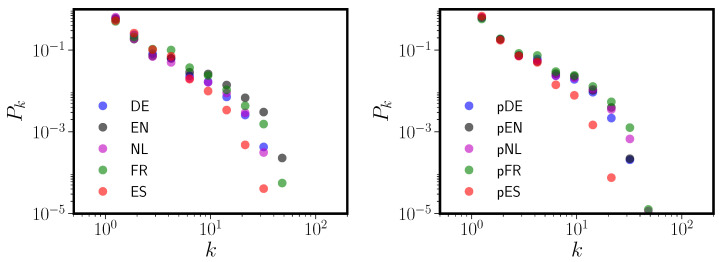
The left panel shows the degree distributions $P_{k}$ versus *k* for the five CLEARPOND PNNs. This is a reprint of the left panel of Figure 4, so that comparisons may be more easily made. The right panel shows degree distributions for pseudo-PNNs, each of which is produced using the UNI model (see Section 3.1) and matched to the target language.

We now compare pseudo-PNNs to real PNNs for all five languages: English, Spanish, Dutch, German, French. For this comparison, we used only the UNI model, since it has a very similar degree distribution to the English PNN $P_{k}$ despite containing almost no information about real language phonology and constraints. In each case, the pseudo-PNN is matched in total corpus size and form length distribution to its target language. The left panel of Figure 7 shows the true degree distributions for the five language PNNs (shown also in Figure 4) and the right panel of Figure 7 shows the pseudo-PNNs using the UNI model. Furthermore, Table 4 shows topological parameters for Spanish, French, German, and Dutch and their matched UNI pseudo-PNNs. We omit English in Table 4 because that information is contained in Table 3.

Figure 7 and Table 4 together show that, as in English, the UNI model is able to come remarkably close in shape and topological properties to the real phonological neighbor networks, despite not resembling the real language’s phonology in any way. In fact, using the JSD, among the five languages English is the worst fit by UNI, with Dutch showing the closest correspondence with the UNI model. The clustering of the five language degree distributions for the pseudo-PNNs mimics that seen in the real PNNs, particularly in the manner in which Spanish is separated from the other languages. Given the way the UNI pseudo-PNNs were constructed, this grouping must be driven entirely by the form length distribution. Table 4 shows that the pseudo-PNNs match their target languages quite well overall, with some properties extremely similar, e.g., their clustering coefficients and degree assortativity.

**Figure 8:**
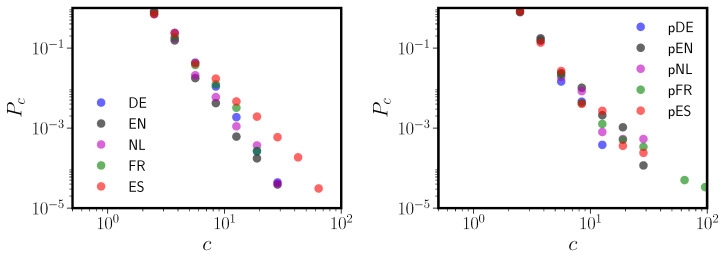
The left panel shows the component size distribution $P_{c}$ versus *c* (compare to Figure 2). The right panel shows component size distributions for pseudo-PNNs, each of which is produced using the UNI model (see Section 3.1) and matched to the target language.

In Figure 2 we showed that the component size distributions for all five language PNNs follow a power law, even moreso than the degree distributions for the PNNs themselves. This has previously only been observed in English and Spanish [22]. However, even these component size distributions do not arise out of any fundamental or universal phonological properties. In the left panel of Figure 8 we reprint Figure 2 to allow easy comparisons. In the right panel we show component size distributions for the five pseudo-PNNs. While the span of $P_{c}$ is somewhat reduced in the pseudo-networks, all the pseudographs clearly have power law size distributions with exponents similar to their target languages. Thus, even the island size distribution is essentially an artifact of the neighbor definition.

### 3.4. Sensitivity to the Form Length Distribution

The previous section demonstrates that the topological properties of phonological neighbor networks constructed using the one-step DAS rule are driven not by any real linguistic feature but by the connection rule itself. While the resulting PNNs are remarkably insensitive to the degree to which real phonological constraints are used in their construction, we have also shown that the PNNs *are* sensitive to the shape of the form length distribution $P_{l}$. In this section, we investigate that sensitivity further. We do that by generating four more UNI lexicons with different input form length distributions and compare the resulting pseudo-PNNs to the FK English database. The four form length distributions are as follows.

- **EMP**. $P_{l}$ is the empirical form length distribution for FK, exactly as in Figure 5 and Table 2.
- **ZTP(1x)**. $P_{l}$ is a zero-truncated Poisson (ZTP) model fit to the empirical distribution. The ZTP distribution has the form
  (2) $$P_{\mathrm{ZTP}}\left(k;\lambda \right)=\frac{\lambda^{k}}{(e^{\lambda }-1)k!},$$
  which assuming independence among the empirical length values $x_{i}$ leads to a model likelihood
  (3) $$L\left(\lambda \right)=\prod_{i=1}^{N}\frac{\lambda^{l_{i}}}{\left(e^{\lambda }-1\right)l_{i}!}$$
  in which λ can be determined via numerical maximization of L(λ).
- **ZTP(1.5x)**. This model is a zero-truncated Poisson model for $P_{l}$ with a mean equal to 1.5 times the mean of the ML λ of ZTP(1x).
- **GEO**. Here $P_{l}$ follows a geometric distribution
  (4) $$P_{\mathrm{GEO}}\left(k;p\right)=p{\left(1-p\right)}^{k-1}$$
  for which the parameter *p* is chosen to make the mean of GEO equal to the mean of the empirical English $P_{l}$.

**Figure 9:**
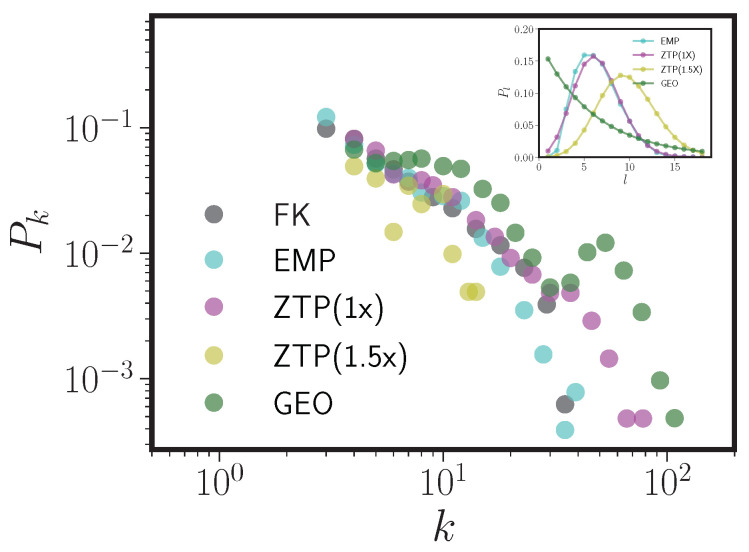
Degree distributions (main panel) for UNI pseudo-PNNs constructed using four different phonological length distributions: the empirical English form length distribution (EMP), a zero-truncated Poisson fit to the empirical distribution (ZTP(1x)), a zero-truncated Poisson with shifted mean (ZTP(1.5x)), and a geometric distribution (GEO) with the same mean as EMP. The real FK network is shown for comparison, and the inset shows the four different form length distributions.

We chose ZTP(1x) as a simple but relatively poor approximation to the form length distributions of the real languages; the real form length distributions are all underdispersed relative to Poisson. ZTP(1.5x) as compared to ZTP(1x) is similar to the difference between the Spanish $P_{l}$ and those of English and French (see Figure 4). GEO is chosen to have an identical average length to English phoneme strings, but otherwise has a shape radically different than any $P_{l}$ we observe. Figure 9 shows degree distributions for pseudo-PNN constructed using UNI pseudolexicons, with each of these four choices for $P_{l}$, along with the degree distribution of FK for comparison. The inset in Figure 9 shows the form length distribution used to produce the pseudolexicon yielding the PNN in the main panel. Table 5 compares the topological properties of those four pseudo-PNNs to FK and each other.

It is clear from Figure 9 that the shape of the PNN degree distribution is extremely sensitive to the form length distribution. The JSD between each model’s degree distribution and that of the real English FK network is closest for UNI plus EMP, and diverges rapidly as $P_{l}$ takes on shapes increasingly unlike EMP. Even the relatively small differences in the shape of EMP and ZTP(1x) lead to large changes in the tail mass of the degree distribution. The difference between the degree distribution of ZTP(1x) and ZTP(1.5x) is similar to the difference between the degree distributions of English or French and Spanish (see Figure 4). In addition, Table 5 shows that the PNN made from ZTP(1.5x) is much smaller (fewer nodes and edges) than any of the other models. This is expected given the reduction in probability of short phonological forms in ZTP(1.5x) when compared to EMP, ZTP(1x), or GEO; the probability that two strings from the UNI pseudolexicon that differ in length by one unit or less are neighbors decays exponentially with string length. Note also from Table 5 that no matter what effect $P_{l}$ has on the degree distribution of the resulting PNN, all graphs show high clustering coefficients, short mean free paths, and high degree assortativity. The size of the giant component is much more variable, which agrees with previous work on the English PNN showing the second moment of the form length distribution strongly influences the giant component size [29] (note the difference in giant component size between ZTP(1x) and ZTP(1.5x)).

## 4. Discussion

We have shown that observed “universal” topological features of phonological neighbor networks [12]—truncated exponential degree distributions, high clustering coefficients, short mean free paths, high degree assortativity and small giant components—are string rather than language universals. That is, inferences from networks based on similarity regarding language ontogeny or phylogeny are suspect, in light of our analyses demonstrating that similar network structures emerge from nearly content-free parameters. One might object to this strong interpretation. The DAS rule obviously captures important relations that predict significant variance in lexical processing due to similarity of phonological forms in the lexicon. Networks based on DAS are able to extend DAS’s reach, as was previously demonstrated with the clustering coefficient [6,7]. Note, though, that clustering coefficient relates to familiar concepts in word recognition that have not been deeply explored in the spoken domain: the notion of neighbors that are friends or enemies at specific positions, discussed by McClelland and Rumelhart in their seminal work on visual word recognition [30]. Consider a written word like *make*, with neighbors such as *take*, *mike*, and *mate*. *Take* is an enemy of the first letter position in *make*, but a friend at all other letter positions, where it has the same letters. A written word with a clustering coefficient approaching 1.0 would have many neighbors that all mismatch at the same position (thus making them neighbors of each other). A word with a similar number of neighbors but a low clustering coefficient (approaching N/L, that is, *N* neighbors evenly distributed of *L* [length] positions) would have more evenly distributed neighbors. For spoken word recognition, the results of Chan and Vitevitch [6] suggest that a high clustering coefficient exacerbates competition because it is heavily loaded on a subset of phoneme positions, creating high uncertainty. In our view, this reveals important details about phonological competition, but not ontogeny or phylogeny of English, or other specifically linguistic structure. Indeed, given the similarity in the distribution of clustering coefficients (among other parameters) in English and in our abstract PNNs, we interpret instances of (e.g.,) high clustering coefficient as string universals rather than language universals.

While phonological neighbor network topology is largely insensitive to the degree of real phonological structure in the lexicon used to construct the neighbor network, we found some amount of sensitivity to the input form length distribution $P_{l}$. Even relatively subtle changes in $P_{l}$ can lead to observable changes in the degree distributions of the resulting neighbor networks, and differences among the five languages we studied here can be almost wholly attributed to differences in form length distributions among the five languages. However, even this sensitivity is only partial. Form length distributions that look nothing like any of the languages we consider here (GEO, although GEO may partially resemble the $P_{l}$ of a language like Chinese), that generate network degree distributions that we do not observe, still yield high clustering coefficients, short mean free paths, and high degree assortativity. The question of what leads to a given language’s $P_{l}$ is a question about language evolution that will be much more difficult to explain, though some parallels might be drawn with work that seeks to understand the evolution of orthography [31,32,33].

At an even deeper level, it may be perilous to attach too much meaning to the topology of any similarity network of phonological forms, at least with respect to human performance in psycholinguistic tasks. This is because these networks do not “do” anything; they have no function. They are not connectionist networks that attempt to model phoneme perception, like TRACE [34] or TISK [35]. No matter how they are constructed, they are basically static summaries of the structure of the speech lexicon; they do not perform a processing function. Insofar as the similarity measure aligns with latency data from human spoken words tasks (e.g., picture naming [7], lexical decision [6], etc.), network properties may encode some features of human performance. While there is evidence that some aspects of human task performance may be predicted from features of neighbor networks [5,6,7,8,9], it is clear from our study that care must be taken in interpreting the results of studies of phonological networks. If the static structure of the lexicon were to be paired with a dynamics that represents mental processing, it would be possible to test the utility of phonological similarity networks for explaining human performance in psycholinguistic tasks.

Vitevitch and his colleagues [36] have done pioneering work in this regard. They introduced the important innovation of diffusion over PNNs to generate time course simulations. However, these time-course simulations differ in crucial ways from the task demands of actual SWR, and the time-course simulations possible with connectionist models like TRACE. Vitevitch et al. [36] used fairly small English DAS graphs (one for each of 24 words, consisting of the word and its 1-hop and 2-hop DAS neighbors, limiting the complexity of possible interactions). Half the words had relatively high and half had relatively low clustering coefficients. Vitevitch et al. [36] compared the rate at which activation spread in graphs with different clustering coefficients. Simulations began with all of the “activity” on the target word. With a positive diffusion coefficient, “activation” spread towards highly connected items. This implements a view of SWR as retrieval, taking seriously the proposal of Chan and Vitevitch [7] that theorists of SWR should reconsider the possibility that the lexicon has robust internal structure that strongly constrains the process of lexical retrieval. This view contrasts sharply with most theories and models of SWR, which focus on the incremental process of mapping a series of consonants and vowels onto possible word forms. The diffusion model and connectionist network models account for different aspects of human SWR. A challenge for future research is determining whether one approach may be able to provide a more comprehensive account of SWR, or whether a novel approach will be required.

## 5. Materials and Methods

### 5.1. Data

We used the freely available online CLEARPOND [20] database to construct DAS-based PNNs for five languages: English, Dutch, German, French, and Spanish. CLEARPOND is described in detail elsewhere [20], but in brief, it includes phonological transcriptions of orthographic forms and frequency information for over 27,000 words from each language. Frequency information for English [37], Dutch [38], German [39], and Spanish [40] is derived from the SUBTLEX database which counts word occurrences in television and movie subtitles. French frequency information is derived from Lexique [41], a fusion of an older French language database (Frantext) with word occurrence information derived from webpages. For all five languages we constructed PNNs based on the DAS rule described above: two words were neighbors and therefore linked with a bidirectional, unweighted edge, if they differed by no more than a single phoneme deletion, addition, or substitution. After PNN construction, we found that, in each language, a significant percentage of the words had no phonological neighbors, ranging from 24% (French) to 45% (Dutch). All singleton words were excluded from any further analysis, since their topological properties are either trivial (e.g., they are all degree zero) or undefined (e.g., the clustering coefficient). In all five languages, the mean length of the neighborless words is larger than that of the words with neighbors, but this difference is not statistically significant (permutation test).

## Figures and Tables

**Table 1:** Topological measures for the five PNNs. Language names are abbreviated using their two-letter ISO language codes: EN (English), NL (Dutch), DE (German), ES (Spanish), and FR (French). *N* is the number of nodes, *m* is the number of edges, $\bar{k}$ is mean degree, **GC size** is the fraction of network nodes that are in the giant connected component, *C* is the clustering coefficient, *l* is mean geodesic path length, α is the power law exponent of the degree distribution, and *r* is the degree assortativity coefficient. Two values occurring in the table with a forward slash denote that quantity computed for the entire graph and only the giant component. Fits to degree distributions were performed via maximum likelihood [21] starting at k=2, except for French which began at k=10. Asterisks denote that the best fitting distribution is not strictly power law but rather truncated power law, as determined via a likelihood ratio test [21].

	EN	NL	DE	ES	FR
*N*	18,983	15,360	17,227	20,728	21,177
*m*	76,092	36,158	41,970	36,111	145,426
$\bar{k}$	8.01	4.71	4.87	3.48	13.7
**GC size**	0.66	0.56	0.58	0.43	0.74
*C* (all/GC)	0.23/0.28	0.16/0.23	0.21/0.24	0.18/0.20	0.24/0.25
*l*	6.68	8.48	8.73	9.41	6.85
α	1.0 *	1.84 *	1.2 *	2.1 *	1.04 *
*r* (all/GC)	0.73/0.70	0.74/0.69	0.75/0.70	0.71/0.62	0.71/0.68

**Table 2:** Topological measures for the FK English corpus and six pseudolexicons matched to it. All rows of the table excepting the final row are as described in Table 1. The final row is the Jensen-Shannon Divergence (JSD) between each model’s degree distribution and the degree distribution of the empirical FK PNN.

	FK	INFT	UNI	CVUNI	CV	SP	PAIR
*N*	7861	1891	2922	1947	3022	3139	4346
*m*	22,745	2841	7501	3532	8687	8811	12,319
$\bar{k}$	5.79	3.0	5.13	3.63	5.74	5.61	5.67
**GC size**	0.69	0.77	0.85	0.80	0.85	0.85	0.87
*C*	0.21	0.19	0.25	0.22	0.27	0.25	0.25
*l*	6.38	7.26	5.34	6.65	5.30	5.40	5.63
α	1.0 *	1.0 *	1.0 *	1.0 *	1.0 *	1.0 *	1.0 *
*r*	0.67	0.60	0.45	0.64	0.48	0.49	0.45
JSD	0.0	0.079	0.011	0.013	0.046	0.0052	0.0086

**Table 3:** Topological measures for the CLEARPOND English corpus (EN) and six pseudolexicons matched to it. All rows of the table are as described in Table 2.

	EN	INFT	UNI	CVUNI	CV	SP	PAIR
*N*	18,252	3192	5911	3942	6219	7098	8705
*m*	59,965	3748	13,281	6857	16,373	18,821	20,922
$\bar{k}$	6.6	2.35	4.49	3.48	5.27	5.31	4.81
**GC size**	0.65	0.36	0.41	0.47	0.38	0.63	0.35
*C*	0.21	0.19	0.25	0.22	0.27	0.25	0.25
*l*	6.81	16.7	6.53	9.43	5.77	10.7	9.35
α	1.0 *	1.0 *	1.0 *	1.0 *	1.0 *	1.0 *	1.0 *
*r*	0.70	0.83	0.74	0.85	0.68	0.71	0.72
JSD	0.0	0.11	0.034	0.030	0.069	0.025	0.046

**Table 4:** Topological measures for four phonological neighbor networks (FR, ES, DE, NL) and matched UNI pseudo-PNNs (pFR, pES, pDE, pNL). All rows of the table are as described in Table 2.

	FR	pFR	ES	pES	DE	pDE	NL	pNL
*N*	12,164	7854	20,018	2198	16,787	4141	14,943	3938
*m*	32,753	21,577	31,812	2852	35,402	8749	31,697	9408
$\bar{k}$	5.38	5.49	3.16	2.60	4.17	4.23	4.24	4.79
**GC size**	0.72	0.36	0.43	0.32	0.57	0.33	0.55	0.57
*C*	0.28	0.27	0.19	0.19	0.21	0.25	0.16	0.27
*l*	7.13	7.49	9.49	9.99	8.88	5.34	8.5	11.59
α	1.0 *	1.0 *	1.0 *	1.0 *	1.0 *	1.0 *	1.0 *	1.0 *
*r*	0.59	0.75	0.70	0.65	0.71	0.67	0.73	0.66
JSD	0.0	0.0080	0.0	0.020	0.0	0.0068	0.0	0.0039

**Table 5:** Topological measures for four UNI pseudo-PNNs (EMP, ZTP-1X, ZTP-1.5X, GEO) and the real FK phonological neighbor network. All rows of the table are as described in Table 2.

	FK	EMP	ZTP(1x)	ZTP(1.5x)	GEO
*N*	7861	2959	2753	211	3592
*m*	22,745	7954	13,127	304	35,938
$\bar{k}$	5.79	5.38	9.54	2.88	20.0
**GC size**	0.69	0.85	0.85	0.64	0.95
*C*	0.21	0.24	0.28	0.19	0.35
*l*	6.38	5.19	4.48	4.71	3.73
α	1.0 *	1.0 *	1.0 *	1.74	1.0 *
*r*	0.67	0.44	0.53	0.46	0.49
JSD	0.0	0.011	0.040	0.086	0.16

**Figure A1:**
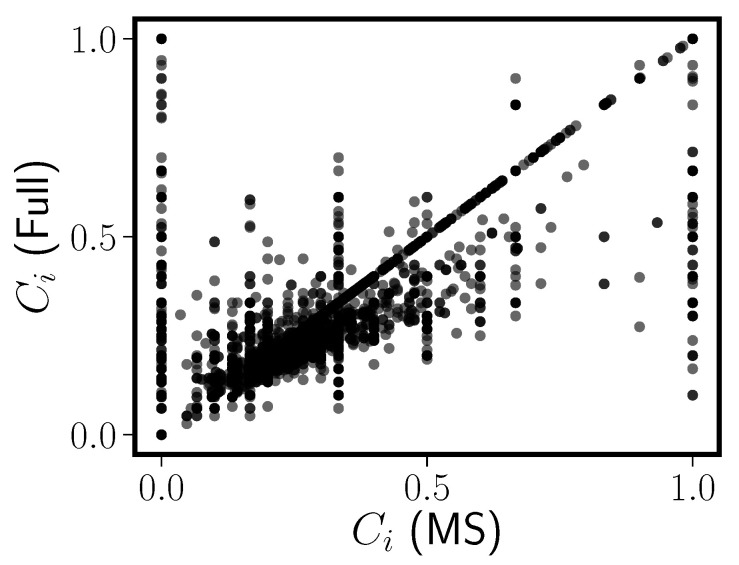
The clustering coefficient for each MS node in the MS graph (*x*-axis) and in the full English CLEARPOND PNN (*y*-axis). The $R^{2}$ of the correlation between the two sets of values is 0.8.

**Figure A2:**
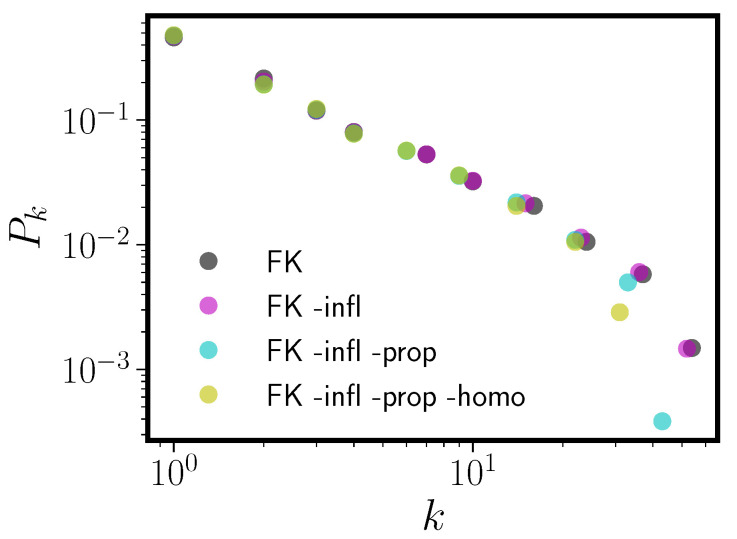
Double log plot of the degree distribution $P_{k}$ against *k* for the FK phonological neighbor network (black circles) when inflected forms (magenta), proper nouns (cyan), and homophones (yellow) are successively removed.

**Figure A3:**
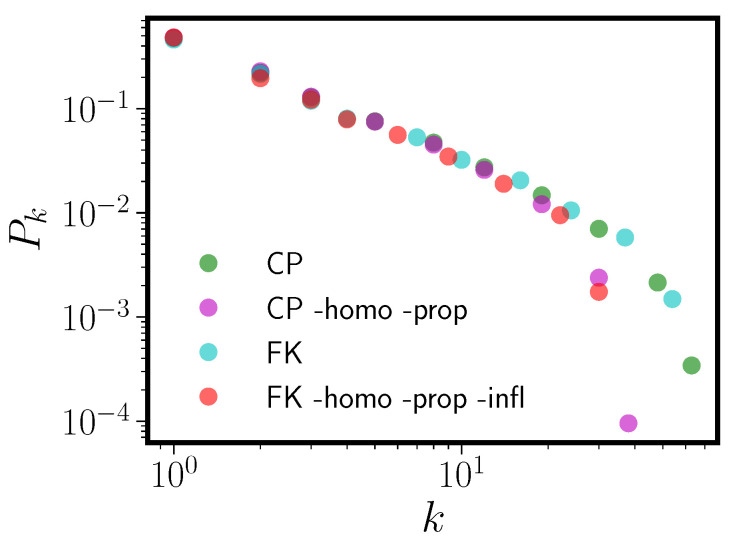
Double log plot of the degree distribution $P_{k}$ against *k* for the CLEARPOND English PNN and FK when various classes of words are removed. The CLEARPOND English PNN degree distribution is shown unaltered (green) and after homophones and proper nouns are removed (magenta), while FK is show unaltered (cyan) and when homophones, proper nouns, and inflected forms are all removed (red).

## Abbreviations

The following abbreviations are used in this manuscript:

NAM	Neighborhood Activation Model
PNN	Phonological Neighbor Network
DAS	Deletion-Addition-Substitution
SWR	Spoken Word Recognition
WS	Watts-Strogatz
BA	Barabasi-Albert
CLEARPOND	Cross-Linguistic Easy-Access Resource for Phonological and Orthographic Neighborhood Densities
FK	Francis and Kucera
EN	English
NL	Dutch
DE	German
ES	Spanish
FR	French
GC	Giant Component
INFT	Infinite Temperature Pseudolexicon
UNI	Noninteracting, Uniform Field Pseudolexicon
CVUNI	Noninteracting, Consonant/Vowel Uniform Field Pseudolexicon
CV	Noninteracting, Consonant/Vowel Field Pseudolexicon
SP	Noninteracting, Spatially Varying Field Pseudolexicon
PAIR	Nearest Neighbor Interactions Pseudolexicon
EMP	Emprical Form Length Distribution
ZTP	Zero-Truncated Poisson Distribution
GEO	Geometric Distribution

## Appendix A. Syllable Level Analysis

To deterimine the number of syllables, we count vowels and dipthongs in the phonological transcription of each word. In addition, we correct for words that end in a phonological “l” with no vowel preceeding the final phoneme. For example, the English word *able* has only a single vowel but is a two-syllable word. We note here that syllable *boundaries* are much harder to determine, but we do not need to decompose the word into its constituent syllables for any of our analysis.

For each language we built two additional graphs: one for the monosyllabic (MS) words only and one for the polysyllabic (PS) words only. Just as in the full PNN, these two new graphs used the DAS rule to determine if two words should be connected by an edge. We show results for English and Dutch in Table A1. Table A1 shows that the topological properties of the PNNs arise by mixing two very different kinds of graphs. For quantities like the clustering coefficient and degree assortativity, this mixing is very mild. The MS graphs tend to cluster more strongly than the PS graphs, and vice versa for degree assortativity, but the differences are not extreme. This is not the case for the rest of the topological measures. Despite having far fewer nodes, the MS graphs have tenfold greater edge density. The MS graphs are almost completely connected; all “islands” in the English and Dutch PNNs are induced by the structure of PS graphs. Mean geodesic paths are quite short in the MS graphs and long in the PS graphs. The MS graphs do not have power law degree distributions at all; that arises due to mixing with the PS graphs (all power-law or truncated power law) in the full graph.

**Table A1:** Topological measures for graphs produced from the CLEARPOND English and Dutch corpora. MS+PS is the full PNN (see also Table 1), MS is a graph formed from only the monosyllabic words, and PS a graph formed from only the polysyllabic words. With the exception of edge density *d* and frequency assortativity coefficient $r_{f}$, all symbols in this table are the same as those in Table 1, and the quantities in the tabhle separated by forward slashes have the same meaning as in Table 1. Edge density is defined as 2m/N(N−1), where *m* is the number of edges and *N* the number of nodes in the graph.

	EN MS + PS	EN MS	EN PS	NL MS + PS	NL MS	NL PS
*N*	18,983	5979	13,004	15,630	2808	12,552
*m*	76,092	50,232	19,808	36,158	16,785	18,396
*d*	0.0004	0.003	0.0002	0.0003	0.004	0.0002
$\bar{k}$	8.01	16.8	3.0	4.71	11.96	2.93
**GC size**	0.66	0.98	0.46	0.31	0.97	0.43
*C*	0.23/0.28	0.3/0.3	0.19/0.26	0.16/0.23	0.31/0.30	0.13/0.20
*l*	6.68	4.63	10.3	4.62	11.8	8.73
α	1.0 *	-	1.04 *	1.84 *	-	1.72
*r*	0.73/0.70	0.65/0.65	0.74/0.66	0.74/0.69	0.59/0.59	0.74/0.65
$r_{f}$	0.104(4) [26σ]	0.068(4) [15σ]	0.089(7) [13σ]	0.126(5) [25σ]	0.055(8) [7σ]	0.100(7) [14σ]

We also compared node-level topology for the MS words in the MS only graph and the full PNN (MS + PS). Most quantities are almost perfectly correlated for these two: these include number of neighbors (degree), number of second neighbors, and eigenvector centrality. All of these quantites are highly correlated with $R^{2}\geq 0.95$. The clustering coefficient for the MS words in the two English graphs is more weakly similar ($R^{2}=0.8$), with large outliers (see Figure A1). It would be interesting to revisit the proposed relationship between node clustering and spoken word recognition [6] facility in light of these findings.

When we performed the same syllable-level calculations for the other three languages in the CLEARPOND database, we find a consistent story (results not shown). In all cases, MS giant component sizes are much larger than PS GC sizes, MS edge densities are close to tenfold larger, and MS mean geodesic path lenghts are much shorter. PS degree distributions are well-fit by truncated power laws and have much more consistent power law exponents than we see for the full PNNs in Table 1. All five languages except Dutch have power law exponents in the range 1.0–1.04. Dutch is better fit by a non-truncated power law with exponent 1.72 (see Table A1). Furthermore, the clustering coefficient and degree assortativity are similar in the MS and PS graphs, just as in English and Dutch. As in English and Dutch, clustering coefficients are larger for the MS graphs in German, Spanish, and French.

## Appendix B. Lexical Issues

In this section, we discuss some lexical issues that are relevant to the pseudolexicon models we construct in Section 3. Table A2 shows the thirteen most highly connected words in English CLEARPOND. What should be clear from Table A2 is that proper nouns (*Lowe*) and homophones (*see*, *sea*) are overrepresented. Our pseudolexicon models directly generate phonological forms with no orthographic tags; we thus cannot represent homophones in our models and must remove them from the PNN graphs for comparison. Another category of words not represented in Table A2 that we cannot easily model is inflected forms, for example word-final “s” for plurals in English. Lemmas and their inflected forms occur much more frequently than expected at random, particular for longer (multisyllabic) words. We discuss the effects of these three categories of words (homophones, inflected forms, and proper nouns) on the resulting PNN degree distribution, and explain our protocol for their removal.

We began with the Francis and Kucera 1982 English corpus [28] (hereafter FK), which consists of the Brown corpus of English words, along with prononuciation (phonological transcription) for every item and an indication as to whether the item is a lemma or an inflected form. We used FK rather than CLEARPOND here because, as shown below, we lack some of this word-level information in CLEARPOND and cannot remove all three categories of words in the CLEARPOND PNNs. We successively removed inflected forms, proper nouns, and homophones from FK as follows.

- **Proper Nouns**. Any word whose orthographic (written) form begins with a capital letter is assumed to be a proper noun. This rule applies equally well to the PNNs for FK and CLEARPOND. However, we emphasize here that because of the rules for capitalization in German (all nouns are capitalized), we cannot systematically remove proper nouns for all five languages in CLEARPOND.
- **Inflected Forms**. FK includes lemma numbers for all the words, so we can simply remove any words that are not lemmas. We do not have this information for any words in CLEARPOND and thus cannot remove them. To try to remove inflected forms in CLEARPOND we could, for example, remove all words with word-final phonological “z”. This would remove English plurals but also improperly remove some lemmas (*size*). Even if this were desirable, we would need different rules for all five languages. Therefore we are forced to keep all inflected forms in the CLEARPOND PNNs.
- **Homophones**. Homophones are items with identical phonological transcriptions but different orthography. These are relatively simple to remove in both FK and CLEARPOND English, and the same procedure works in any language. We search the nodes for sets of items with identical phonological transcriptions. For example, *see* and *sea* would comprise one homophone set in English, and *lieu*, *loo*, and *Lou* another. One of the items from each homophone set, chosen at random, is kept in the PNN and the nodes corresponding to all other items in the set are deleted.

**Table A2:** The thirteen words in English CLEARPOND with the highest degree. Note the prevalence in this list of (i) proper nouns and (ii) homophones (e.g., *see*, *sea*).

Word	Degree
Lea	68
Lee	68
Lew	66
loo	66
lieu	66
Lai	63
lye	63
lie	63
Lowe	62
low	60
male	60
see	60
sea	60

Figure A2 shows the degree distribution of the FK PNN when inflected forms, proper nouns, and homphones were successively removed. Two features of this figure deserve mention. First, the main effect of these classes of words is in the tail of the degree distribution. Secondly, removal of inflected forms causes very little change compared to removal of proper nouns and homophones. It is relatively easy to understand why the largest changes to the degree distribution occur at large *k*, at least for homophones. Consider a single orthographic form *w* that is also a homophone with degree *d*. All of the other orthographic forms in its homophone set are connected to both *w* and all of the *d* neighbors of *w*. If there are *N* words in the homophone set, we end up with *N* nodes each with degree *d* + N−1. Thus, homophone sets can boost the degree of both their neighbors (since a neighbor of one is a neighbor of all other words in the set) and the homophones themselves. As an example, a homophone set of size 10 in which one of the words has 10 neighbors yields 10 nodes with degree 19. Removing members of the homophone set will therefore tend remove nodes of large degree and therefore shift the tail of $P_{k}$.

Figure A3 compares removal in English CLEARPOND to FK. We first note that, despite being based on completely different corpora, the unaltered English CLEARPOND and FK yield similar PNNs. In addition, as in FK, removal of homophones and proper nouns in CLEARPOND tends to truncate the tail of the degree distribution. As we noted above, the only class of words that we can consistenly remove from all five CLEARPOND languages is homophones, and we remove these for all model comparisons. The number of homophone sets, mean set size, and the number of nodes removed from the graph when the removal procedure described above is implemented, for each of the five languages in CLEARPOND, is shown in Table A3. Table A3 indicates that for all languages except French, the majority of homophone sets are pairs like *see* and *sea*.

**Table A3:** Number of homophone sets $N_{H}$, mean homophone set size $\mu_{H}$ and the number of nodes removed from the CLEARPOND PNNs when homophones are removed. Note the wide variation in the number of homophones across the five languages.

Language	$N_{H}$	$\mu_{H}$	Nodes Removed
EN	731	2.09	795
DE	440	2.10	485
ES	1059	2.03	1123
FR	9013	2.63	14,735
NL	417	2.08	449
